# Significant Variability in Terpene Content and Secondary Organic Aerosol Formation Potential from Ozonolysis of Consumer Product Categories Used Indoors

**DOI:** 10.1021/acsestair.5c00380

**Published:** 2026-04-01

**Authors:** Sofie K. Schwink, Maximilian J. Schmid, Jonathan M. Silberstein, Marina E. Vance

**Affiliations:** † Environmental Engineering Program, 1877 University of Colorado Boulder, 1111 Engineering Drive, Boulder, Colorado 80309, United States; ‡ Department of Mechanical Engineering, University of Colorado Boulder, 1111 Engineering Drive, Boulder, Colorado 80309, United States

**Keywords:** SOA formation potential, volatile chemical products, limonene SOA, VCP composition

## Abstract

Measuring the secondary organic aerosol (SOA) formation potential of common consumer chemical products is important for understanding the impacts of product use on ambient air quality. We performed controlled chamber experiments measuring SOA yields from reactions of ozone with common consumer products from three categories: perfumes, deodorants, and cleaning liquids. SOA mass yields ranged between 0.2% and 7% with no seed in the chamber and between 3% and 17% with inorganic seed in the chamber. The average yield across all experiments was 4%. We used GC-MS to measure the concentration of several terpenes in our tested products. Limonene concentrations in the VCPs ranged from 0.1% to 0.3% and beta-pinene concentrations ranged from 0.4% to 0.7%. The effective density (ρ_eff_) of SOA formed via heterogeneous nucleation onto ammonium sulfate seed ranged from 1.47 ± 0.01 g cm^–3^ to 1.58 ± 0.14 g cm^–3^. The ρ_eff_ of SOA formed via homogeneous nucleation exhibited more variation, ranging from 1.08 ± 0.02 g cm^–3^ to 1.49 ± 0.03 g cm^–3^. Our results demonstrate significant differences in the composition and SOA formation potential of common VCP categories.

## Graphical abstract

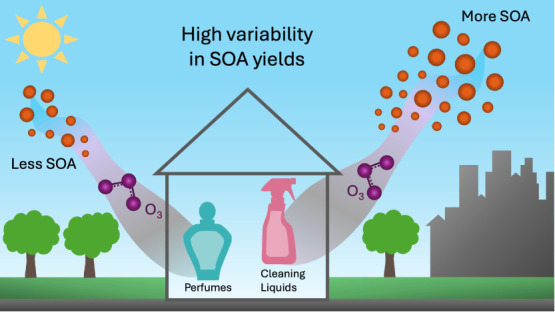

## 1 Introduction

Secondary organic aerosols (SOA), which are formed by the multistage oxidation of volatile organic compounds (VOC), are a significant air pollutant around the world.^1,2^ In the United States, the use of volatile chemical products (VCPs), such as cleaning agents, personal care products (PCPs), and paints, now contributes to over half of ambient SOA precursor concentrations in industrialized cities.^3^ Industrial and mobile source emissions have been reduced in recent decades with the implementation of improved control technologies,^4,5^ so the relative importance of SOA from VCPs has increased. Exposure to SOA is associated with increased cardiorespiratory disease mortality,^6^ oxidative stress, and immune system dysfunction,^7^ and may lead to up to 340,000 PM_2.5_-related premature deaths each year around the world.^8^ Because of this, it is important that we characterize how a variety of VCPs and VCP components contribute to SOA formation in both controlled laboratory and real-world studies.

VCPs are widely used in homes around the world. Seltzer et al.^9^ estimate a VOC emission rate from VCP use in the United States of 9.5 kg person^–1^ year^–1^, which equates to 6.4 kg of carbon emissions person^–1^ year^–1^. In 2016, this meant a total of 3.05 Tg of organic emissions were released from VCP use in the US. In their study, ∼76% of VCP emissions came from cleaning products, personal care products, and paints and coatings. McDonald et al.^3^ performed a mass balance of organic compounds across the US petrochemical industry in 2012 and found that total VOC emissions to the atmosphere from VCP use in industrialized cities were more than twice as high as those from the transportation sector. The mass of oil and natural gas used as fuel was ∼15 times higher than the mass used as chemical feedstocks to produce VCPs, but VCPs generally have higher emission factors than fuels, emitting more VOCs to the atmosphere per mass of product used. Because of this, SOA formation potential is higher for VCPs than for fuel emissions.

VCPs contribute more significantly to total VOC concentrations in more densely populated areas. In New York City, 78% of anthropogenic VOC emissions come from VCPs, whereas in Boulder, Colorado, VCP use contributes to 42% of anthropogenic VOC emissions.^10^ Qin et al.^11^ estimate that after constraining SOA mass yield to 5% in a model, emissions from VCPs contribute to ∼41% of photochemical organic PM_2.5_ in Los Angeles in the summer. SOA formation potential in different cities is affected by many factors, including population density and the type of urban environment. Shah et al.^12^ collected urban oxidation flow reactor (OFR) measurements to quantify SOA formation potential in different types of urban environments, including an urban low-rise, a street canyon, and an industrial area. They observed approximately three times as much morning-time SOA formation potential in the street canyon as in the low rise. Monoterpenes, commonly associated with the presence of trees and scented chemical products, were found in higher concentrations in the street canyon than in the other environments, even though the low-rise environment had denser tree cover. The enhancement in monoterpene emissions in the street canyon was likely from the use of VCPs in this densely populated environment.

Monoterpenes are often used as a tracer for fragrances and are among the most common and reactive VOCs associated with VCP emissions.^13^ Rogers et al.^14^ estimate that 46–59% of monoterpenes in downtown Chicago are of anthropogenic origin, largely from VCP use. Seltzer et al.^15^ found that monoterpene emissions from VCPs positively correlate with population density in New York City, Pittsburgh, Chicago, and Denver and are dominated by limonene. Concentrations of other compounds commonly found in VCPs also correspond with population density and can be used as tracers of product use in densely populated urban areas. Decamethylcyclopentasiloxane (D5-siloxane) is associated with PCPs, monoterpenes with fragrances, p-dichlorobenzene with insecticides, D4-siloxane with adhesives, para-chlorobenzotrifluoride with solvent-based coatings, and Texanol with water-based coatings.^13^ In addition to these tracer compounds, most VCPs contain other ingredients that may contribute to SOA formation. Little research has been done investigating how specific chemical products available to consumers, which may contain a wide variety of reactive compounds, contribute to SOA formation.

VOCs in the atmosphere can react with a variety of oxidants to produce SOA. The main oxidants participating in these reactions are OH and ozone.^16^ OH is highly reactive with a wide variety of compounds. Ozone is reactive with gas-phase compounds that contain double bonds, and these compounds often react more with ozone than with OH.^17^ Differences in ratios of ozone to OH, along with concentrations of other oxidants, impact SOA formation potential and chemistry. Many experimental studies have measured SOA formation potential from reactions of VCP components with OH because of its prevalence in the atmosphere and reactivity with a wider range of compounds than ozone.^12,16,18–21^ Several studies have investigated SOA formation from reactions of ozone with different individual chemical compounds, including squalene,^22^ various individual terpenoids and terpenoid mixtures,^23^ and a variety of monoterpenes, including limonene.^24,25^ Malashock et al.^26^ estimate that the annual global average outdoor ozone concentration in urban areas is 51 ppb. At these concentrations, ozone is an important contributor to urban SOA formation. Although ozone is reactive with fewer compounds than OH, it is an important oxidant in the atmosphere, and products of its reactions with VCPs should be investigated. In addition to oxidant type and concentration, SOA formation potential is affected by the concentration and composition of background seed particles.

A few studies have investigated the effects of seed particles on SOA formation via homogeneous and heterogeneous nucleation. For example, Lambe et al.^27^ observed SOA yields from OH oxidation of isoprene 3–5× higher in the presence of sulfate seed particles than in the absence of seed. Heterogeneous nucleation, the condensation of vapor onto existing condensation nuclei, occurs more readily in the atmosphere than homogeneous nucleation because it can occur at very low vapor saturation ratios. Homogeneous nucleation, the formation of particles from supersaturated vapor in the absence of condensation nuclei, occurs less frequently due to the increased level of supersaturation required (vapor saturation ratios ∼2–10).^28^ However, homogeneous nucleation is easily observed in a laboratory, and performing experiments without seed helps us understand more about VCP reactivity than if we studied heterogeneous nucleation reactions alone.

In the past decade, many studies have investigated SOA formation from specific VCP components, such as limonene. Advances in instrumentation, like the development of the OFR, have driven this research forward. The OFR, which operates by rapidly oxidizing precursor gases at very high oxidant concentrations in a flow-through chamber, allows for fast measurements of the maximum aerosol mass that a set of precursor gases can produce.^29^ It allows for fast determination of aerosol yields, and its use has become increasingly popular in the last two decades. More research is needed to investigate how real consumer products may contribute to SOA formation in controlled laboratory environments like chambers and OFRs and in the real world. The goal of this study was to quantify SOA formation potential from reactions of ozone with three common categories of VCP: perfumes, deodorants, and cleaning liquids. We performed chamber experiments with and without inorganic seed and tested products that are commonly found in stores and affordable for consumers (<$25/product). While the few products tested here are not representative of all products in each of these categories, this study demonstrates the significant variability in SOA formation potential between and within common VCP categories.

## 2 Materials and Methods

### 2.1 Product Selection

We investigated SOA formation from two VCPs in each of three different categories, for a total of six products. The product categories tested were perfumes, deodorants, and cleaning liquids. Products within each category were chosen based on their popularity, ease of access in stores, affordability (<$25/product), and potential for reactivity with ozone. Detailed information on all products tested can be found in Table S1. We also performed experiments investigating SOA formation from limonene, chosen because it is a common component of scented VCPs and because reactions of limonene with ozone have been well characterized in the literature.^30–32^

### 2.2 Experimental Setup

For all experiments, we ozonated a 0.68 m^3^ stainless steel chamber to 6.5 ± 0.6 ppm using a UV ozone generator (M610, Jelight, Irvine, CA) with 0.5 lpm of dry air passing through it. Ozone concentrations were monitored throughout each experiment using an ozone monitor (202, 2B Technologies, Broomfield, CO). For heterogeneous nucleation experiments, we introduced 91 ± 11 μg m^–3^ of ammonium sulfate ((NH_4_)_2_SO_4_) seed to the chamber through 1.8 m (6’) of 6.35 mm (0.25”) inner diameter (i.d.) conductive tubing using an atomizer equipped with a 0.46 m (1.5′) diffusion dryer. The atomizer contained a solution of 1 g of (NH_4_)_2_SO_4_ in 200 mL of ultrapure water and was connected to the chamber for 50 s at a pressure of 60 psi for heterogeneous nucleation experiments. Self-indicating silica beads in the diffusion dryer were dried in an oven for 2 h at 135 °C after every few experiments. The average initial seed diameter was 45 ± 5 nm, and the average seed surface area was 3970 ± 280 μm^2^ cm^–3^. Figure S1 shows a representative size distribution of the seed aerosol. The average temperature in the chamber across all experiments was 22.4 ± 0.8 °C, and the average relative humidity was 7.5 ± 2.2%.

We introduced the VCP of interest by putting ∼3 g of it into a beaker and then placing the beaker in an airtight 2.8 L stainless steel container and passing 3.5 lpm of clean, dry air through it via 1.5 m (5′) of 6.35 mm (0.25”) i.d. Teflon tubing. This allowed products to enter the chamber in the gas phase. The air flowing into this system passed through an activated carbon filter to remove VOCs before entering the stainless steel container. After introducing the VCP for 30 min, we switched a valve to deliver clean air to the chamber through 1.5 m (5′) of 6.35 mm (0.25”) i.d. Teflon tubing for a 60 min decay period. We weighed the VCP before and after introducing it to the chamber and used the mass difference in our calculation of SOA yield. For limonene experiments, 0.4 μL of limonene was pipetted into a beaker and allowed to completely evaporate into the chamber.

We performed control experiments using each VCP and limonene with no ozone in the chamber. We also performed controls in an ozonated chamber with ultrapure water instead of a VCP. Control experiments were performed for each condition with and without (NH_4_)_2_SO_4_ seed. We did not observe new particle formation during these control experiments. During seeded control experiments, (NH_4_)_2_SO_4_ seed particles coagulated over time, growing from an initial D_p_ of 45 ± 5 nm to 87 ± 9 nm after 1 h in the chamber.

### 2.3 SOA Yield and Effective Density Measurements

We used a scanning mobility particle sizer (SMPS 3082, TSI, Shoreview, MN), equipped with a long differential mobility analyzer (DMA 3081, TSI) and a butanol-based condensation particle counter (CPC 3750, TSI) to monitor particle concentrations and size distributions throughout each experiment. Size distributions were measured every 3 min.

We used an aerosol particle mass analyzer (APM 3602, Kanomax, Andover, NJ) with a long DMA (3081, TSI) upstream and a water-based CPC (3788, TSI) downstream to measure the mass of 60, 80, and 100 nm particles, which we used to calculate aerosol effective density (ρ_eff_) using a method similar to Schwink et al.^33^ Density measurements were averaged over a 10 min period of data collection for each size bin (30 min total). There were generally no significant size-dependent differences in ρ_eff_, so we used the average ρ_eff_ value across the three size bins to convert particle number concentrations measured by the SMPS to mass concentrations for our yield calculations. A schematic of our experimental design is shown in Figure 1. All SOA yield and ρ_eff_ measurements were performed three times per product, both with and without (NH_4_)_2_SO_4_ seed.

**Figure 1:**
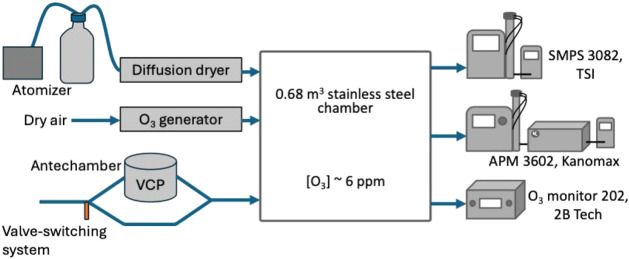
Experimental design for SOA formation experiments. Experiments were performed for each VCP with and without seed in the chamber.

Aerosol mass yield (Y) was calculated using eq 1:

1 $$Y=\frac{m_{\mathrm{aerosol}}}{m_{\mathrm{product}}}$$

where m_aerosol_ is the total aerosol mass generated during an experiment, and m_product_ is the mass of the product that evaporated during the experiment. Only a portion of m_product_ is expected to react with ozone to form SOA, so it is important to distinguish that these yields were calculated based on the mass of the product that evaporated, not the mass that actually participated in reactions with ozone. We calculated SOA yields this way, rather than by calculating using the product mass that participated in reactions, because it allows for direct comparison of sales volumes of these products to the potential for SOA formation in the atmosphere. This makes these results more applicable to the real world. For heterogeneous nucleation experiments, the mass of the seed was subtracted from the total aerosol mass for our yield calculations. Size-resolved particle wall losses were characterized and added to our yield calculations. We did not characterize VOC wall losses. For these calculations, we assumed that particles were perfectly spherical, so our calculated SOA yield values represent the upper limit of the actual aerosol formation that occurred.

### 2.4 VCP Composition Measurements

We assessed the composition of the six VCPs we tested using a gas chromatograph–mass spectrometer (GC-MS, 6090N GC, 5975 MS, Agilent Technologies, Santa Clara, CA). For this analysis, we used 490 μg of the solid deodorant samples, 1 μL of the perfumes, and 5 μL of the cleaning liquids. These masses were chosen because they provided a strong signal to the instrument without saturating the MS detector. Samples were extracted for organics analysis following the methodology outlined in Silberstein et al.^34^ Briefly, samples were spiked with 25 μL of an internal standard before extraction and were washed twice with 10 mL of dichloromethane solvent for 15 min with ultrasonic agitation in a water bath. Extracted samples were then evaporated to 2 mL under a gentle stream of nitrogen. Quadratic calibration curves were generated using a high-concentration terpene mixture (Spex Certiprep) and were paired with a deuterated acenaphthene internal standard for quantification. Assessed uncertainties were solely a function of calibration curve uncertainty. During our measurements, the internal standard spike occurred prior to the extraction process such that any analyte losses during extraction led to corresponding losses in the internal standard, so we did not account for uncertainty due to analyte losses. Volumetric concentrations for liquid samples were converted to mass concentrations (g g-sample^–1^) by assuming a density of the VCP samples of 1 g mL^–1^.

## 3 Results and Discussion

### 3.1 SOA Yields of VCPs

We observed SOA formation from all VCPs immediately after their introduction to the chamber, and the average yield value across all experiments was 0.044 ± 0.045 g g^–1^. Representative timeseries of particle number concentration, mass concentration, and heat maps for homogeneous and heterogeneous nucleation experiments with Cleaning Liquid 2 are shown in Figures S2–S3. SOA mass yields in g of aerosol formed per g of product evaporated for homogeneous nucleation experiments ranged from 0.002 ± 0.000 g g^–1^ to 0.07 ± 0.01 g g^–1^. Yields for heterogeneous nucleation experiments were significantly higher than homogeneous nucleation yields for the same products and ranged from 0.03 ± 0.00 g g^–1^ to 0.17 ± 0.01 g g^–1^. SOA yields from homogeneous and heterogeneous nucleation experiments with all VCPs are shown in Figure 2. Yield values for all products are shown in Table S2.

**Figure 2:**
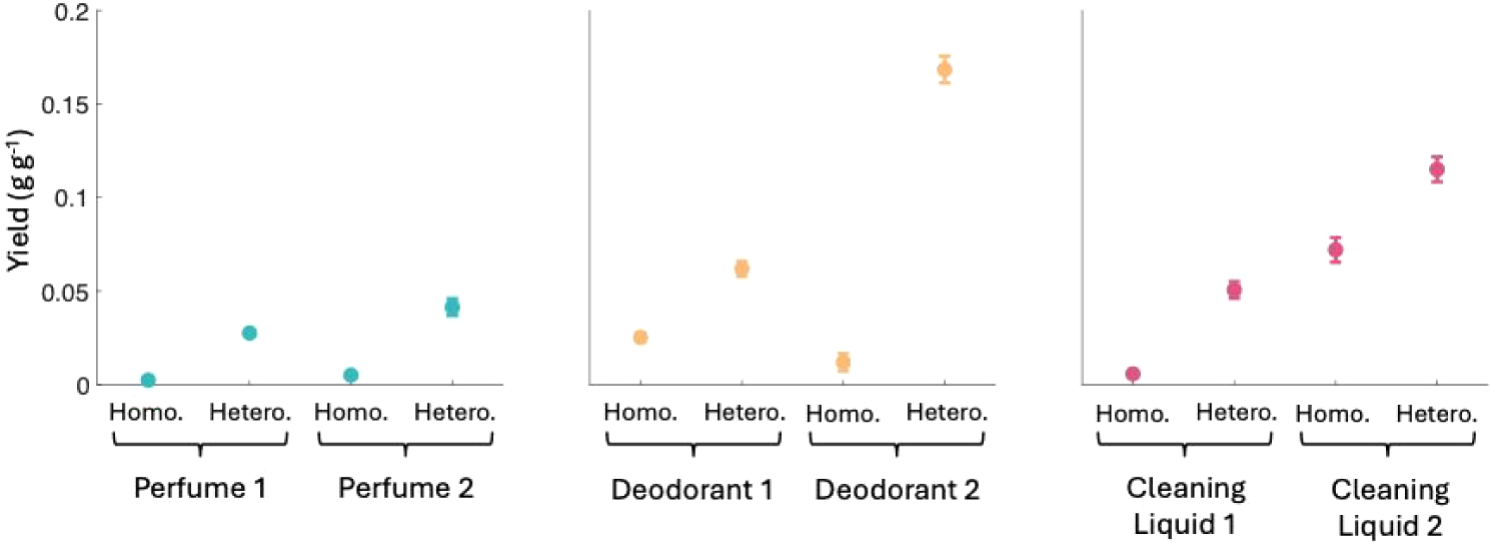
SOA yields from homogeneous and heterogeneous nucleation experiments for all VCPs tested. Averages are shown ± standard deviation (*n* = 3 for each condition).

There is little information in the literature on the SOA yields of specific consumer products. Models are often relied upon for determining SOA yields of consumer product categories because they can simulate more realistic environmental conditions than those created in an OFR or in a chamber like the one used for this study. However, models often make assumptions about SOA mass yields, and assumptions vary between studies. In their model of reactive organic carbon emissions from VCPs, Seltzer et al.^9^ use an effective SOA yield of 0.053 g g^–1^ for VCPs, which approximates the overall average seen in our study (0.044 ± 0.045 g g^–1^). In their mass balance of VOC emissions from the US petrochemical industry, McDonald et al.^3^ use an SOA yield of 0.064 ± 0.015 g g^–1^ for cleaning agents, very similar to the average of 0.061 ± 0.041 g g^–1^ for our study, and of 0.025 ± 0.006 g g^–1^ for personal care products, about half of what we observed for perfumes and deodorants (0.045 ± 0.053 g g^–1^). Some studies have measured SOA yields of individual VCP tracer compounds. For example, Lambe et al.^27^ measured SOA yields for monoterpenes (fragrances) of 0.3 ± 0.1 g g^–1^. The concentration of these compounds in VCPs is generally well below 100%, since there are typically many ingredients in VCPs. Because of this, we should expect yields from consumer products to be lower than yields from tracer compounds. Figure S4 shows the homogeneous SOA yields as a function of limonene and beta-pinene content of VCPs used in this study. Monoterpenes are generally reactive with ozone, but some VCP tracer compounds, like D5 siloxane, are not reactive with ozone. This makes it difficult to compare our experimental yield values from reactions with ozone to experimental data from reactions with OH. We performed several experiments with D5 in our chamber, during which we did not observe SOA formation.

Many factors influence experimental SOA yields, including background seed particle concentration,^35^ oxidant type and concentration,^29^ relative humidity,^36^ variability in VCP composition and reactivity,^37^ and interactions with other compounds in ambient air.^38^ The presence or absence of background seed particles can have significant impacts on SOA yields. Lambe et al.^27^ observed SOA yields from isoprene ∼3–5× higher from heterogeneous nucleation than from homogeneous nucleation in reactions with OH. Our heterogeneous nucleation yields were ∼1.5–14× higher than homogeneous nucleation yields for the same products. This variability could be due to different effects of seed particles on the amount of vapor supersaturation required for condensation to occur. SOA yields may be lower than their potential maximum values due to the condensation of vapors onto chamber walls. Metal chambers have been characterized as vapor sinks in literature.^29^

Our measured SOA yields generally fall into the expected range based on the literature. There is significant variation in the composition of VCPs within and between product categories, and our selected products are not representative of the full range available to consumers. Measuring SOA yields of actual consumer products, rather than of their individual chemical components, is useful in gaining insight into the range of SOA yields possible from real indoor activities. Comparing our yields from real consumer products to SOA yields for well-characterized compounds, such as limonene, allows us to draw better comparisons between our results and results from other studies.

### 3.2 SOA Yields of Limonene

Unlike our tested VCPs, pure limonene SOA yields were approximately the same for heterogeneous and homogeneous nucleation experiments, at 0.53 ± 0.07 g g^–1^ with seed and 0.52 ± 0.06 g g^–1^ without seed. Distinct “banana” plots show that limonene SOA was formed via homogeneous nucleation even with seed in the chamber (Figures S6 and S7). This may have occurred due to the rapid oxidation of gas-phase limonene molecules, the quick formation of peroxy radicals, and the subsequent formation of very low-volatility dimers at very high ozone concentrations.^39^ We did not monitor the chemical speciation of reaction products during these experiments, so we are unable to confirm whether this occurred during limonene experiments or whether this reaction pathway was taken by the limonene in our tested VCPs.

SOA yields from reactions of limonene with ozone have been well characterized in the literature. Northcross and Jang^31^ measured a yield of 0.49 ± 0.05 g g^–1^, Lee et al.^32^ measured 0.58 ± 0.01 g g^–1^, and Saathoff et al.^30^ measured 0.35 g g^–1^. Our limonene SOA yields fall into the range of yields from literature. Limonene, which is a monoterpene, is commonly used as a fragrant additive in VCPs. Both cleaning liquids used in this study had limonene included on their ingredients. Manufacturer-provided ingredient lists for all VCPs used in this study are in Table S3.

Many VCP manufacturers do not provide complete ingredient lists for their products, often listing “fragrance” as an ingredient without specifying which compounds are included in the fragrant components of the products. To better understand which compounds in our VCPs may have contributed to SOA formation, we quantified the concentrations of limonene, alpha-pinene, and beta-pinene in all tested VCPs.

### 3.3 Limonene and Beta-Pinene Content of VCPs

Concentrations of limonene, alpha-pinene, and beta-pinene were quantified for each VCP using GC-MS. Alpha-pinene masses were all below the lower bound of the calibration curve, so these results are not reported. Alpha-pinene is the largest contributor to biogenic SOA precursors and is used in some VCPs as a fragrant additive.^40^ Chromatograms from our GC-MS measurements for all six tested VCPs are shown in Figures S8–S13. Mass ratios for each sample are shown in Table 1.

**Table 1:** Limonene and Beta-Pinene Content of VCPs Measured with GC-MS, in g of Compound per g of Product^a^

Product	Limonene (g g^–1^)	Beta-pinene (g g^–1^)
Perfume 1	0.0010 ± 0.0001	0.0011 ± 0.0002
Perfume 2	0.0022 ± 0.0003	0.0010 ± 0.0002
Deodorant 1	0.0013 ± 0.0002	0.0013 ± 0.0003
Deodorant 2	0.0012 ± 0.0002	0.0065 ± 0.0011
Cleaning liquid 1	0.0023 ± 0.0003	0.0016 ± 0.0003
Cleaning liquid 2	0.0026 ± 0.0003	0.0004 ± 0.0001

a Averages are shown ± calibration curve uncertainty.

Limonene mass concentrations ranged between 0.0010 g g^–1^ and 0.0026 g g^–1^. Beta-pinene concentrations varied more, between 0.0004 g g^–1^ and 0.0065 g g^–1^. Elevated mass concentrations of a quantified terpene did not correlate with enhanced concentrations of other terpenes, as ratios of limonene to beta-pinene ranged between 0.18–6.15, and alpha-pinene values were below the lower limit of our calibration curve regardless of the mass of limonene and beta-pinene. This study provides an example of significant variability in the terpene content and SOA formation potential of a few products. More products would need to be tested to understand the range and variability of terpene concentrations in common VCPs. There is little information in the literature or online regarding the terpene content of VCPs.

We plotted the monoterpene content of VCPs versus heterogeneous SOA yields for all tested products (Figure 3) and generally observed increases in heterogeneous SOA yields with increases in total monoterpene content, suggesting that monoterpenes were a significant factor driving SOA formation from these products.

**Figure 3:**
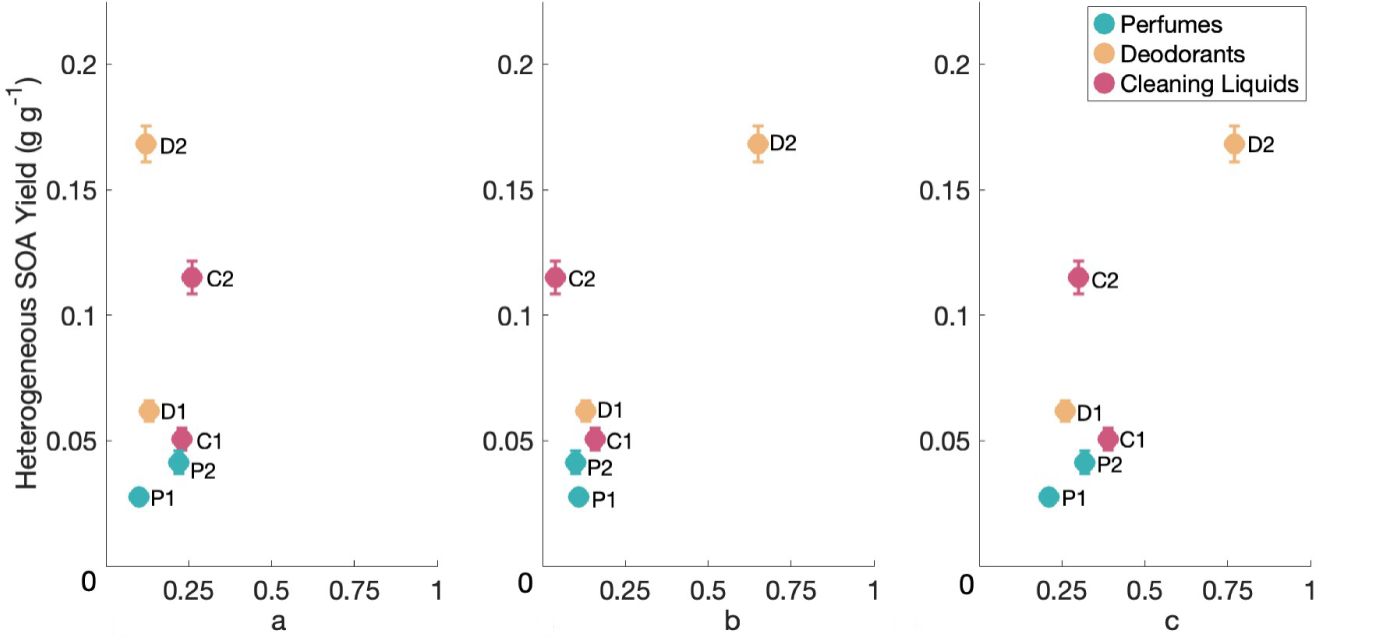
(a) VCP limonene content, (b) beta-pinene content, and (c) the sum of limonene and beta-pinene content versus SOA yields from heterogeneous nucleation experiments.

To determine whether limonene and beta-pinene in our tested VCPs formed SOA via homogeneous nucleation when there was seed in the chamber, like we observed during our limonene experiments, we plotted the limonene and beta-pinene content of VCPs versus the ratio of the average SOA yield with seed and without seed (Figure S5). Generally, with the exception of Deodorant 1, SOA yield ratios decreased with increasing VCP limonene content. This suggests that SOA production from the oxidation of limonene in VCPs took place primarily via homogeneous nucleation, like we observed in pure limonene experiments. The same relationship was not observed for beta-pinene, with yield ratios varying from 1.61 to 14.1, and no clear dependence on beta-pinene concentration of the product. Beta-pinene’s only double bond is exocyclic, so its reaction with ozone tends to produce less oxygenated fragmentation products, which are likely more volatile and less able to form SOA via homogeneous nucleation than the oxidation products formed from limonene ozonolysis.^39^ Additionally, the lifetime of limonene in these experiments is shorter than the lifetime of beta-pinene based on rate coefficients, which means homogeneous nucleation is more likely to occur for limonene than beta-pinene. Using the rate coefficients for reactions of ozone with limonene and beta-pinene from Atkinson and Arey,^17^ an ozone concentration of 6.5 ppm, and atmospheric pressure in Boulder, CO of 630 Torr, the lifetimes of limonene and beta-pinene are 0.6 and 8.3 min, respectively. Based on the decay rates of limonene and beta-pinene at the ozone concentration in our chamber, we expect that all reactions between monoterpenes and ozone were complete in the chamber by the end of each experimental period.

By combining information on the mass of limonene in each VCP and our measured limonene SOA yields, we can estimate how much limonene SOA formed from each product. We can use this calculation to estimate how much of the total SOA formed during homogeneous and heterogeneous nucleation VCP experiments came from reactions of limonene within the VCPs. We estimated limonene SOA formation from VCPs between 0.00053 g g^–1^ and 0.00138 g g^–1^ for homogeneous nucleation experiments and between 0.00052 g g^–1^ and 0.00135 g g^–1^ for heterogeneous nucleation experiments. Values for all homogeneous and heterogeneous nucleation experiments for all VCPs are shown in Table S4. From this information and our measured SOA yields of VCPs, we can also estimate how much limonene contributed to total SOA formation during experiments. Limonene contributed to between 1.9% and 26.5% of homogeneous SOA formation and between 0.4% and 2.8% of heterogeneous SOA formation. All estimates of limonene contributions to total SOA formation are in Table S5. These values suggest that limonene contributed significantly to SOA formation from all tested VCPs, but that other compounds were also important in these reactions, especially during heterogeneous nucleation experiments.

### 3.4 SOA Effective Density

The average ρ_eff_ of SOA formed via heterogeneous nucleation onto (NH_4_)_2_SO_4_ seed across our three measured size bins ranged from 1.38 ± 0.06 g cm^–3^ to 1.58 ± 0.14 g cm^–3^. These values were similar to the ρ_eff_ of (NH_4_)_2_SO_4_ seed alone (1.51 ± 0.02 g cm^–3^). ρ_eff_ of SOA formed via homogeneous nucleation exhibited more variation, with size-averaged values ranging from 1.08 ± 0.02 g cm^–3^ to 1.49 ± 0.03 g cm^–3^. For all products except for Deodorant 1 and limonene, the ρ_eff_ of SOA formed via heterogeneous nucleation was higher than the ρ_eff_ from homogeneous nucleation. Differences in ρ_eff_ between homogeneous and heterogeneous nucleation experiments were statistically significant. Average ρ_eff_ values for SOA from experiments with VCPs, limonene, and (NH_4_)_2_SO_4_ seed are shown in Table 2. Size-resolved ρ_eff_ values from all experiments are shown in Tables S6–S7.

**Table 2:** ρ_eff_ of SOA Formed from VCPs via Heterogeneous and Homogeneous Nucleation, Measured by the APM^a^

	Heterogeneous	Homogeneous
Product	ρ_eff_ (g cm^–3^)	ρ_eff_ (g cm^–3^)
Perfume 1	1.49 ± 0.01	1.08 ± 0.02
Perfume 2	1.51 ± 0.00	1.15 ± 0.03
Deodorant 1	1.47 ± 0.01	1.49 ± 0.03
Deodorant 2	1.52 ± 0.02	1.16 ± 0.03
Cleaning liquid 1	1.55 ± 0.02	1.17 ± 0.02
Cleaning liquid 2	1.58 ± 0.14	1.36 ± 0.07
Limonene	1.38 ± 0.06	1.43 ± 0.06
(NH_4_)_2_SO_4_ seed (no SOA)	1.51 ± 0.02	-

a ρ_eff_ of limonene SOA and (NH4)_2_SO_4_ seed is also shown. Averages are shown ± standard deviation.

The overall average SOA ρ_eff_ measured for this study was 1.53 ± 0.06 g cm^–3^ with seed and 1.24 ± 0.15 g cm^–3^ without seed. Because ρ_eff_ measurements require an assumption that particles are perfectly spherical, our density value for (NH_4_)_2_SO_4_ seed (1.51 ± 0.02 g cm^–3^) is lower than the material density of ammonium sulfate (1.77 g cm^–3^). Zhou et al.^41^ use a density value of 1.18 g cm^–3^ for VCP-derived SOA based on measurements using a time-of-flight aerosol chemical speciation monitor and an SMPS. Malloy et al.^42^ measured SOA density from alpha-pinene ozonolysis and m-xylene photooxidation and determined density values of 1.24 ± 0.03 g cm^–3^ and 1.35 ± 0.03 g cm^–3^, respectively. Kostenidou et al.^43^ measured SOA density from limonene ozonolysis of 1.49 ± 0.24 g cm^–3^ without seed and 1.56 ± 0.10 g cm^–3^ with seed. Our measured ρ_eff_ values from VCPs fall into the same range as literature values for VCPs and limonene.

Nakao et al.^44^ took measurements of the ρ_eff_ of SOA formed via homogeneous nucleation of 22 different VOC precursors. They found that reactants with higher carbon numbers formed SOA with lower ρ_eff_ than reactants with lower carbon numbers. For example, β-caryophyllene (15 carbons) SOA had a density of 1.22 g cm^–3^, while phenol (6 carbons) SOA had a density of 1.43 g cm^–3^. This trend in decreasing density with increasing precursor size was consistent for different levels of oxidation of the parent molecule and with different oxidants. Kuwata et al.^200^ developed a semiempirical relationship between elemental ratios and organic material density and predict that SOA precursors with longer carbon chains will have lower O/C ratios and form SOA with lower density than precursors with shorter carbon chains. Among our homogeneous nucleation experiments, limonene SOA had the second highest ρ_eff_, after Deodorant 1 SOA. These results suggest that some SOA precursors in almost all our tested VCPs have higher carbon numbers than limonene (10 carbons). The compounds we quantified using GC-MS all contain ten carbons, so we cannot make a definitive statement about whether carbon number and ρ_eff_ are related for this study.

### 3.5 Implications, Limitations, and Recommendations

This study provides insight into how much SOA may form from actual consumer products. SOA yields were consistently higher for each tested VCP during experiments with inorganic seed than without seed and varied between and within product categories. Homogeneous SOA yields ranged from 0.002 to 0.07 g g^–1^, and heterogeneous SOA yields ranged from 0.03 to 0.17 g g^–1^. Limonene SOA formed via homogeneous nucleation regardless of whether there were seed particles in the chamber and fell within the range commonly reported in literature. The limonene and beta-pinene content of VCPs were 0.0010–0.0026 g g^–1^ and 0.0004–0.0065 g g^–1^, respectively. The ρ_eff_ of SOA formed via homogeneous nucleation was 1.08–1.49 g cm^–3^, significantly lower than the heterogeneous nucleation ρ_eff_, which was 1.47–1.58 g cm^–3^.

Our results for SOA yields and SOA ρ_eff_ generally fall within the range reported in literature, but further investigation would be useful to contextualize our results. A deeper analysis of the chemical composition of VCPs would allow us to better compare our measured yield values to yield values for VCP components reported in the literature. We were unable to quantify vapor wall losses, so our calculations likely underestimate SOA formation potential. This underestimation is likely more significant for homogeneous nucleation experiments than for heterogeneous nucleation due to increased wall effects with no seed present.^45^

Analysis of more products would be useful to obtain more representative results for SOA yields and ρ_eff_ values, given the variability we observed in this study. The few products tested in this study are not representative of the full pool of cleaning products and personal care products in the consumer market. There is variation in product types and use patterns within the US and in other countries, which contributes to differences in SOA formation potential between regions. Substantial numbers of individual products would need to be tested to gain a better understanding of the full range of SOA formation potentials of VCPs in different regions. Measuring SOA formation from these products under more realistic indoor and outdoor environmental conditions would also help contextualize our results. Testing a variety of oxidants at different concentrations and with more variation in seed type and concentration would provide a more representative example of how SOA may form in the real world.

This work could help inform us about which categories of VCP would be most helpful to target with regulations. It could also inform consumers about which products may have the most significant impact on indoor and outdoor air quality. Given the significant potential health impacts of exposure to SOA, it is important that more research is done investigating SOA formation, properties, and impacts.

## Supplementary Material



## Notes

Supporting Information includes descriptions of products used, their ingredients, and resulting GC-MS chromatograms, size distributions of ammonium sulfate seed particles, representative heat maps of homogeneous and heterogeneous nucleation experiments, SOA yields for all experiments. Also included are estimated contributions of limonene to total SOA formation via homogeneous and heterogeneous nucleation and size-resolved effective density values for homogeneous and heterogeneous nucleation experiments (PDF)
