# Supplementary material for: Significant Variability in Terpene Content and Secondary Organic Aerosol Formation Potential from Ozonolysis of Consumer Product Categories Used Indoors
Source: ACS EST Air. 2026 Apr 1;3(4):981–9. doi: 10.1021/acsestair.5c00380 (PMC13077635; doi:10.1021/acsestair.5c00380)
Supplement: Supplementary file 1 [file ea5c00380_si_001.pdf]

Supplemental Information for

Significant Variability in Terpene Content and Secondary Organic Aerosol Formation Potential from Ozonolysis of Consumer Product Categories Used Indoors

Sofie K. Schwink[a], Maximilian J. Schmid[a], Jonathan M. Silberstein[b], Marina E. Vance[a,b]*

[a]Environmental Engineering Program, University of Colorado Boulder, 1111 Engineering Drive, Boulder, CO 80309, USA.

[b]Department of Mechanical Engineering, University of Colorado Boulder, 1111 Engineering Drive, Boulder, CO 80309, USA.

*Email: marina.vance@colorado.edu

List of figures

Figure S1. Representative size distribution of ammonium sulfate seed particles.

Figure S2. Timeseries of particle number concentration, mass concentration, and banana plot for a homogeneous nucleation experiment with Cleaning Liquid 2.

Figure S3. Timeseries of particle number concentration, mass concentration, and banana plot for a heterogeneous nucleation experiment with Cleaning Liquid 2.

Figure S4. VCP terpene content versus homogeneous SOA yields.

Figure S5. VCP terpene content versus heterogeneous SOA yields.

Figure S6. Banana plot for a homogeneous nucleation experiment with limonene.

Figure S7. Banana plot for a heterogeneous nucleation experiment with limonene.

Figure S8. GC-MS chromatogram for Perfume 1.

Figure S9. GC-MS chromatogram for Perfume 2.

Figure S10. GC-MS chromatogram for Deodorant 1.

Figure S11. GC-MS chromatogram for Deodorant 2.

Figure S12. GC-MS chromatogram for Cleaning Liquid 1.

Figure S13. GC-MS chromatogram for Cleaning Liquid 2.

List of tables

Table S1. List of products used for experiments.
Table S2. SOA yield values for all experiments.
Table S3. Ingredient lists of VCPs reported by manufacturers.
Table S4. Estimated contributions of limonene to total SOA formation.
Table S5. Potential contribution of limonene to total SOA formation.
Table S6. Size-resolved ρ_{eff} values for homogeneous nucleation experiments.
Table S7. Size-resolved ρ_{eff} values for heterogeneous nucleation experiments.

Table S1. Chemical products used for experiments.

Product	Description
**Perfume 1**	Proper Cologne Prescott Birchwood + Tonka
**Perfume 2**	Mix Bar Cloud Musk Hair and Body Mist
**Deodorant 1**	Old Spice Classic Original Deodorant
**Deodorant 2**	Dove 0% Aluminum Deodorant Stick Pomegranate & Lemon Verbena
**Cleaning liquid 1**	Mrs. Meyers Clean Day Multi-Surface Everyday Cleaner Lemon Verbena
**Cleaning liquid 2**	Method All-Purpose Cleaner Lime + Sea Salt

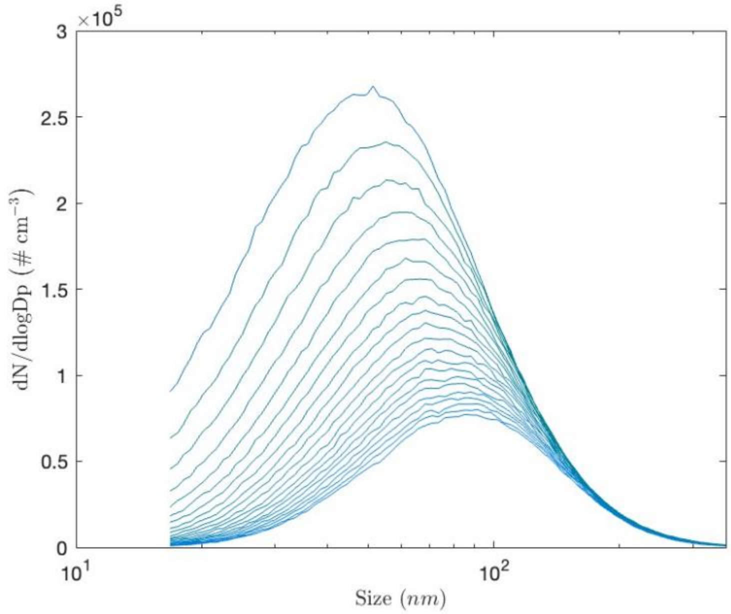

Figure S1. Representative seed size distributions for ~1 h after seed injection. Size distributions are three minutes apart.

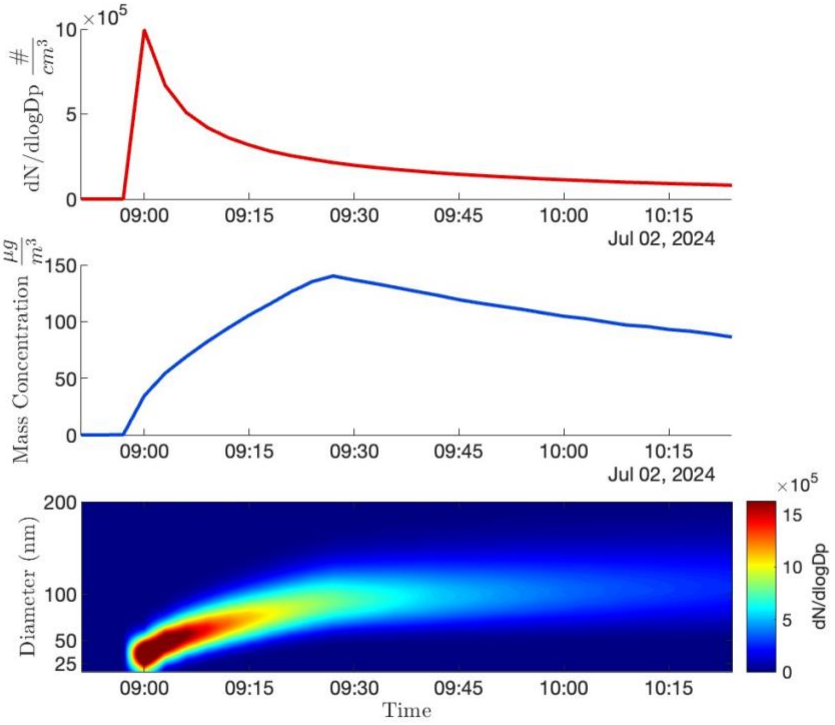

Figure S2. Particle number concentration, mass concentration, and banana plot for a homogeneous nucleation experiment with Cleaning Liquid 2.

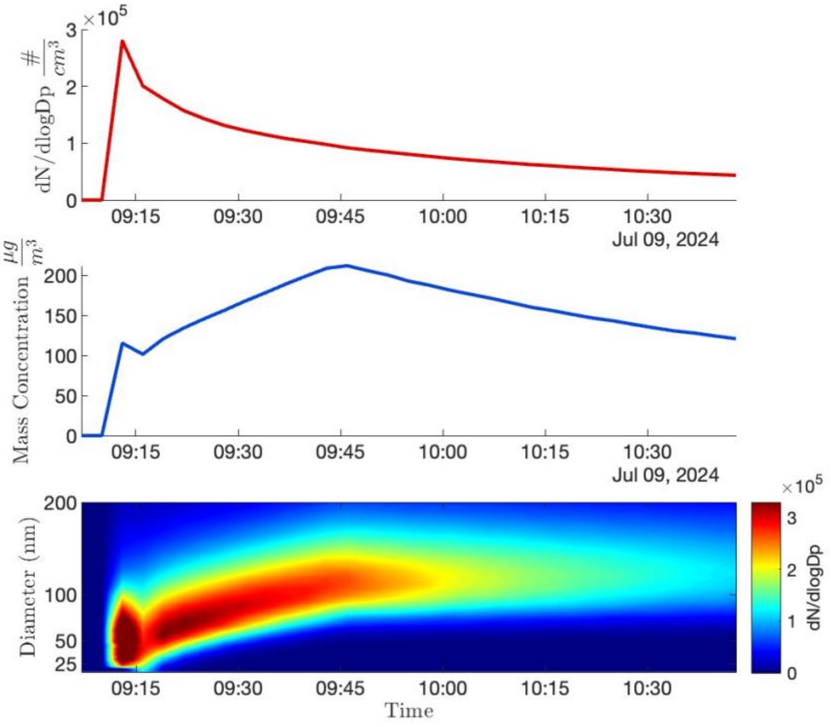

Figure S3. Particle number concentration, mass concentration, and banana plot for a heterogeneous nucleation experiment with Cleaning Liquid 2.

Table S2. SOA yields from all experiments. Averages are shown ± standard deviation (n = 3 for each condition).

Product	Homogeneous Yield (g g^{-1})	Heterogeneous Yield (g g^{-1}
**Perfume 1**	0.002 ± 0.000	0.028 ± 0.001
**Perfume 2**	0.005 ± 0.001	0.041 ± 0.004
**Deodorant 1**	0.025 ± 0.002	0.062 ± 0.004
**Deodorant 2**	0.012 ± 0.005	0.170 ± 0.007
**Cleaning liquid 1**	0.006 ± 0.001	0.051 ± 0.005
**Cleaning liquid 2**	0.072 ± 0.007	0.120 ± 0.007
Average among VCPs	*0.020 ± 0.025*	*0.069 ± 0.048*
**Limonene**	0.520 ± 0.060	0.530 ± 0.070

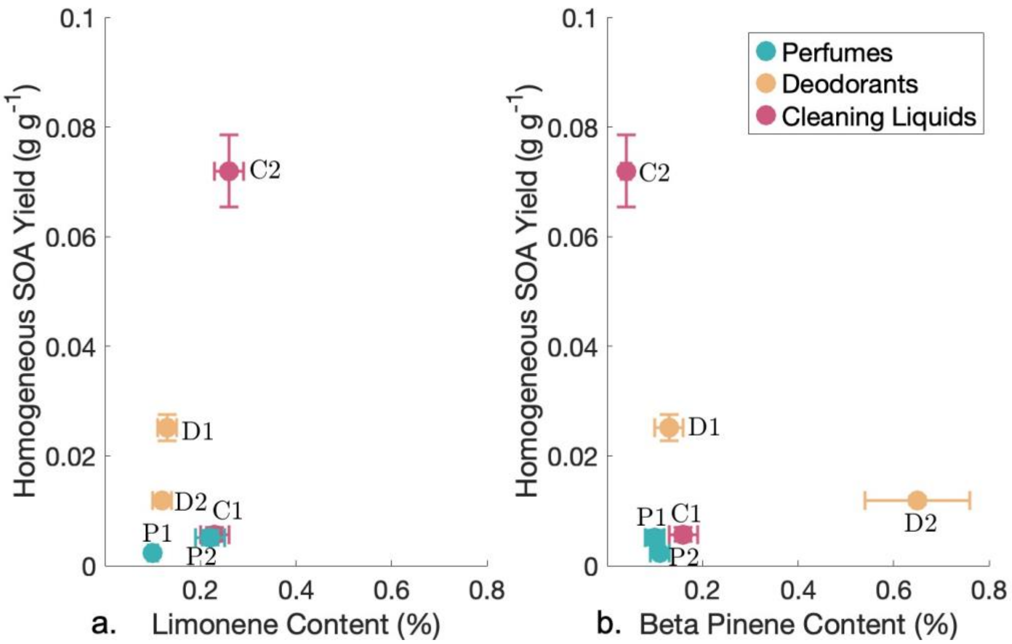

Figure S4. (a.) VCP limonene content and (b.) beta-pinene content versus SOA yields from homogeneous nucleation experiments.

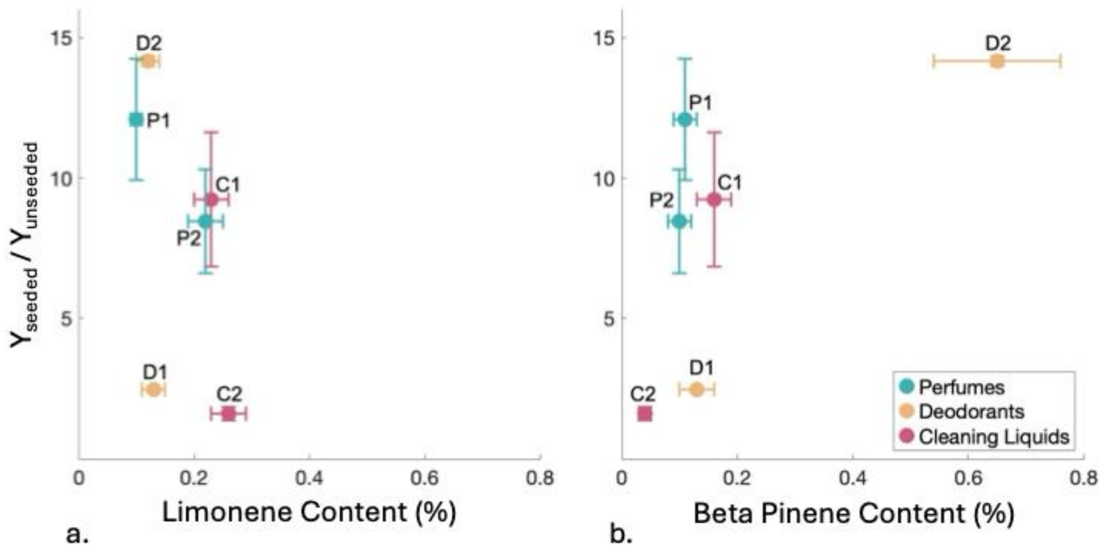

Figure S5. Ratios of SOA yields from experiments with seed (Y_{seeded}) to SOA yields from experiments without seed ($Y_{unseeded}$) versus (a.) VCP limonene content and (b.) VCP beta pinene content. Averages are shown ± standard deviation. Individual products are labeled next to their datapoints.

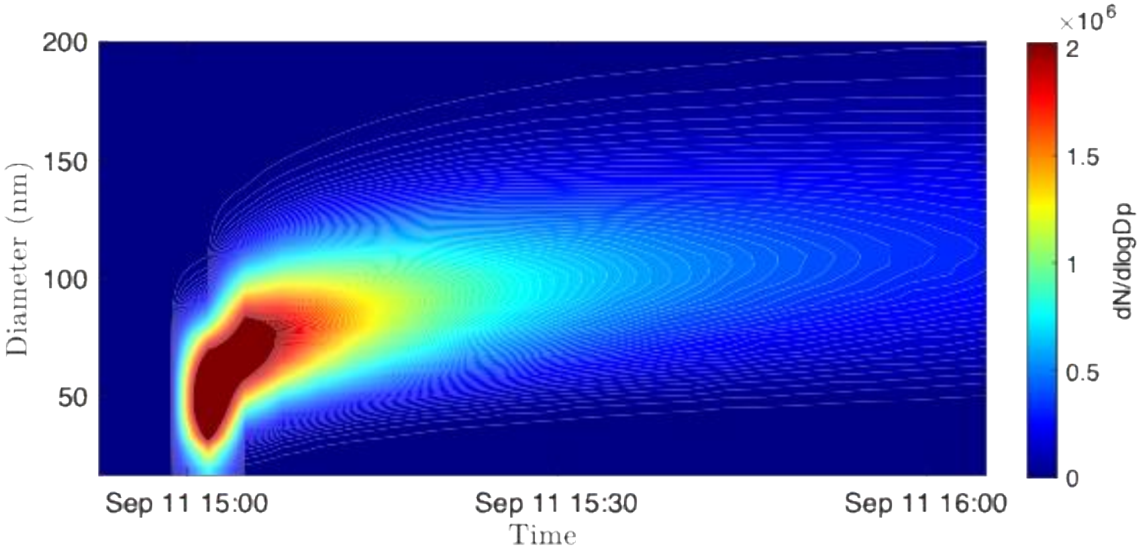

Figure S6. Heat map ("banana plot") from a limonene experiment without seed.

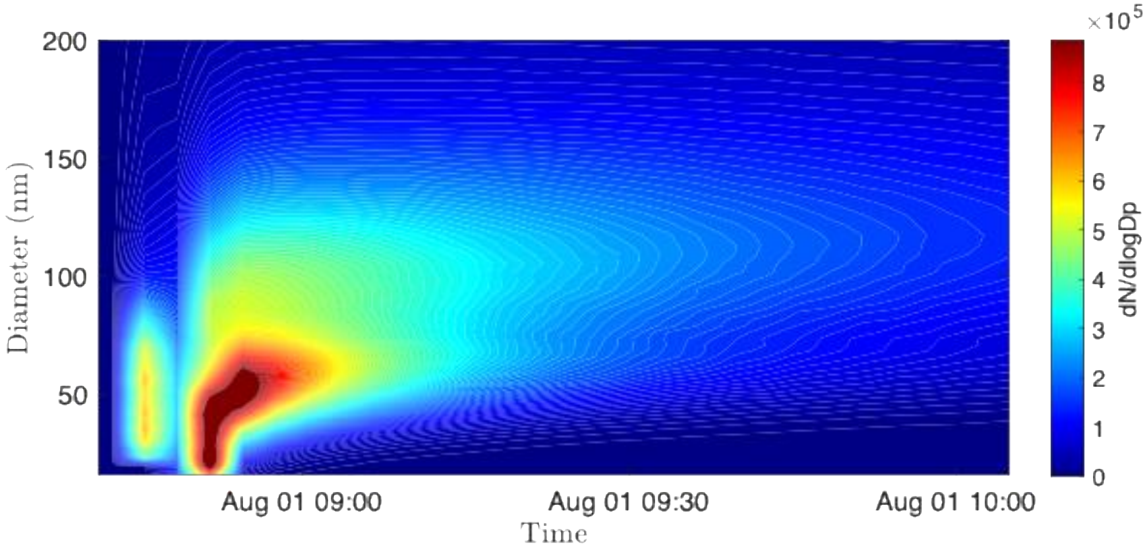

Figure S7. Heat map ("banana plot") from a limonene experiment with seed.

Table S3. Ingredient lists for all VCPs as reported by the manufacturers.

Product	Ingredients
**Perfume 1**	Alcohol Denat., Fragrance, Water
**Perfume 2**	Alcohol Denat., Water (Aqua/Eau), Fragrance (Parfum), Ethylhexyl Methoxycinnamate, Butyl Methoxydibenzoylmethane, Ethylhexyl Salicylate, Red 4 (Ci 14700), Yellow 5 (Ci 19140), Violet 2 (Ci 60725)
**Deodorant 1**	Alcohol Denatured, Propylene Glycol, Sodium Stearate, Water, Fragrance, Tetrasodium EDTA, Yellow 10, Green 5
**Deodorant 2**	Dipropylene Glycol, Water (Eau), Glycerin, Propylene Glycol, Sodium Stearate, Poloxamine 1307, Fragrance (Parfum), Stearic Acid, Disodium EDTA, BHT, Simethicone, Benzyl Alcohol, Benzyl Salicylate, Citronellol, Coumarin, Hexyl Cinnamal, Limonene, Linalool
**Cleaning liquid 1**	Water, Decyl Glucoside, Fragrance, (Alpha-lsomethyl-lonone, Benzyl Benzoate, Eugenol
**Cleaning liquid 2**	Water, Decyl Glucoside, Lauryl Glucoside, SodiumCarbonate, Sodium Gluconate, Laureth-7 (Ethoxylated Coconut Oil), Citric Acid, Potassium Hydroxide, Fragrance (Benzyl Salicylate, Citral, Hexyl Cinnamal, Limonene), Polymeric Blue, Polymeric Yellow

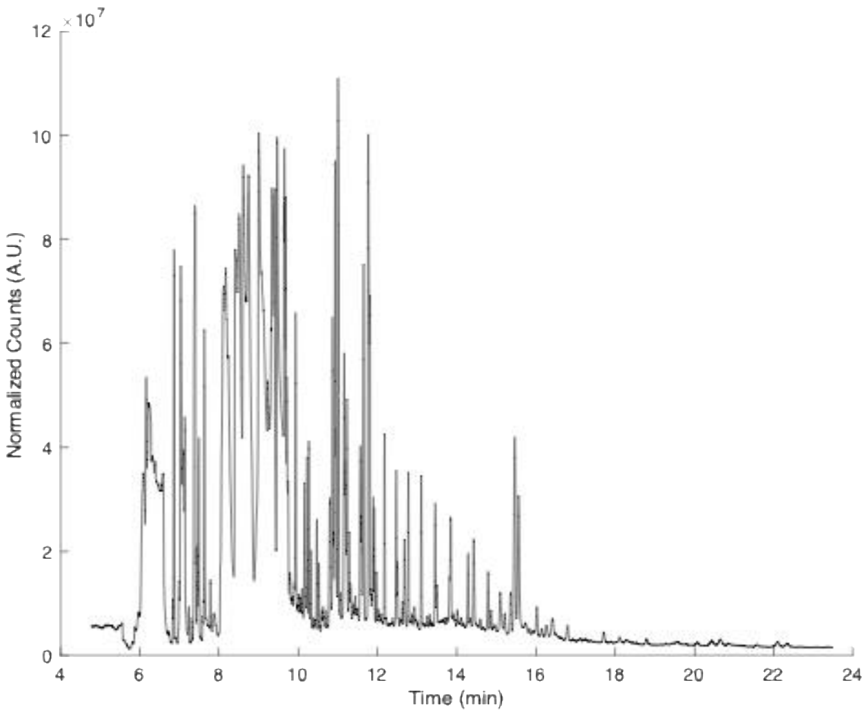

Figure S8. GC-MS chromatogram for Perfume 1.

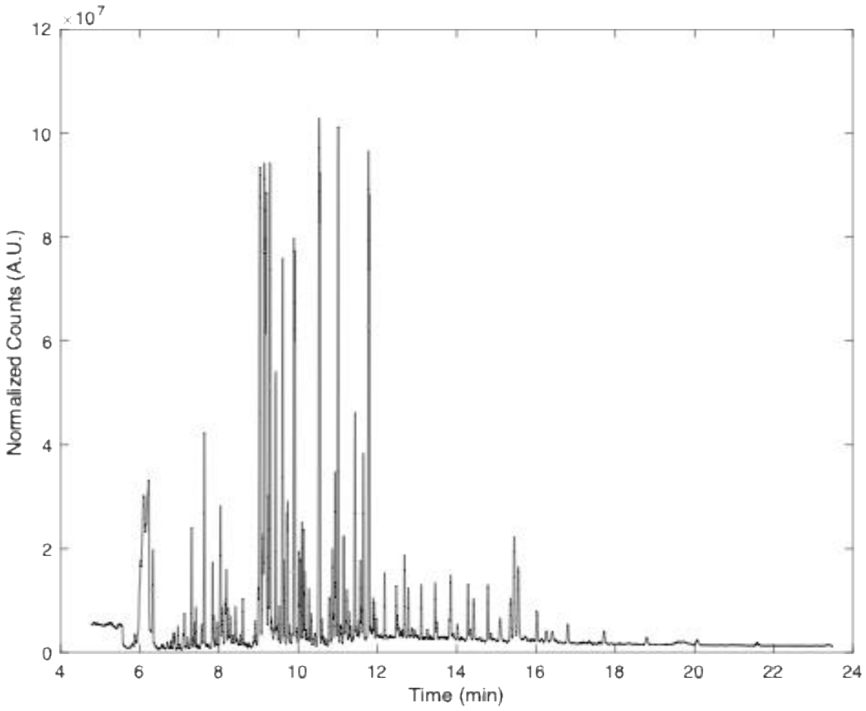

Figure S9. GC-MS chromatogram for Perfume 2.

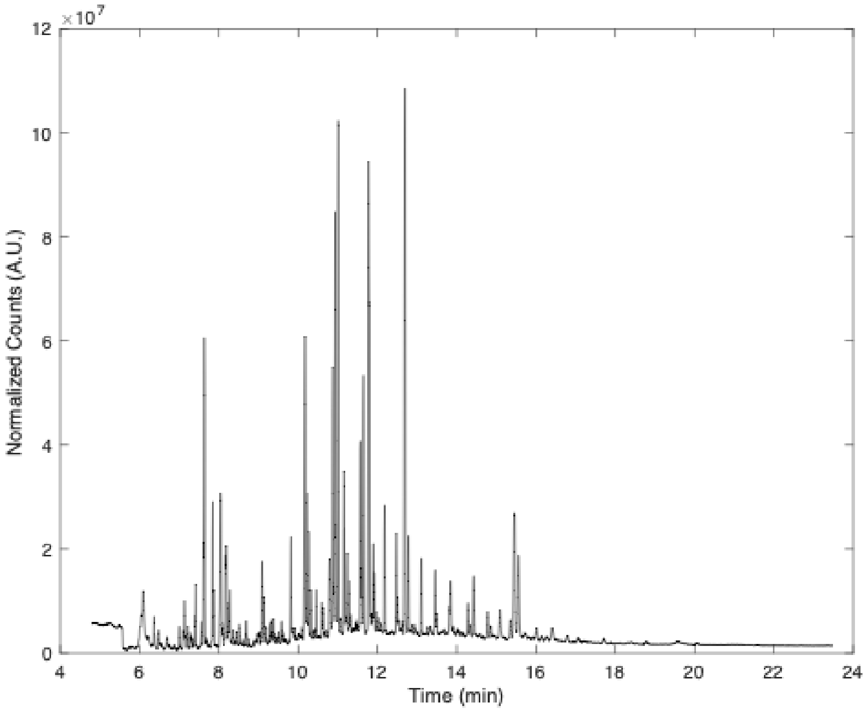

Figure S10. GC-MS chromatogram for Deodorant 1.

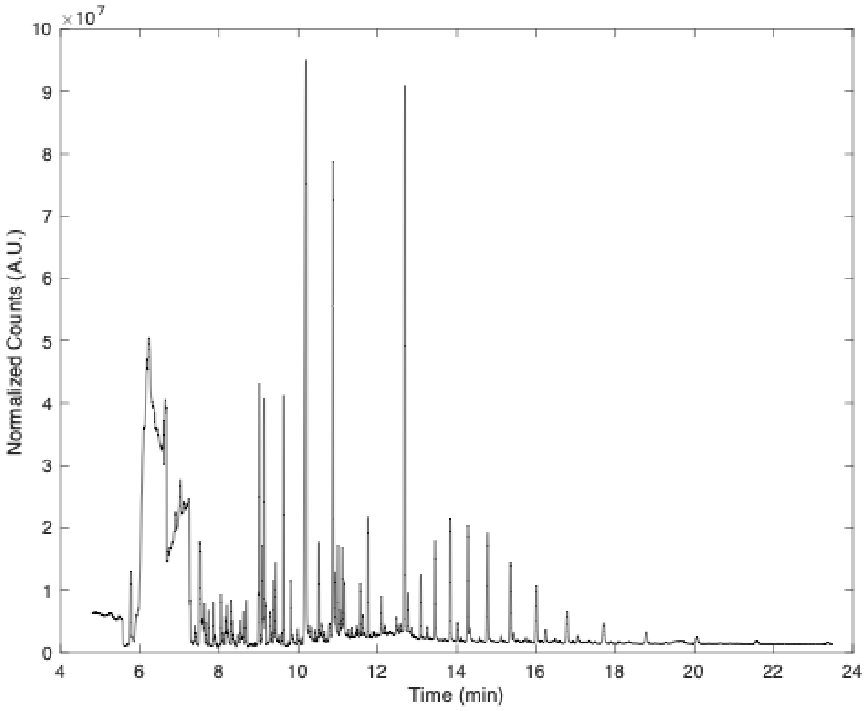

Figure S11. GC-MS chromatogram for Deodorant 2.

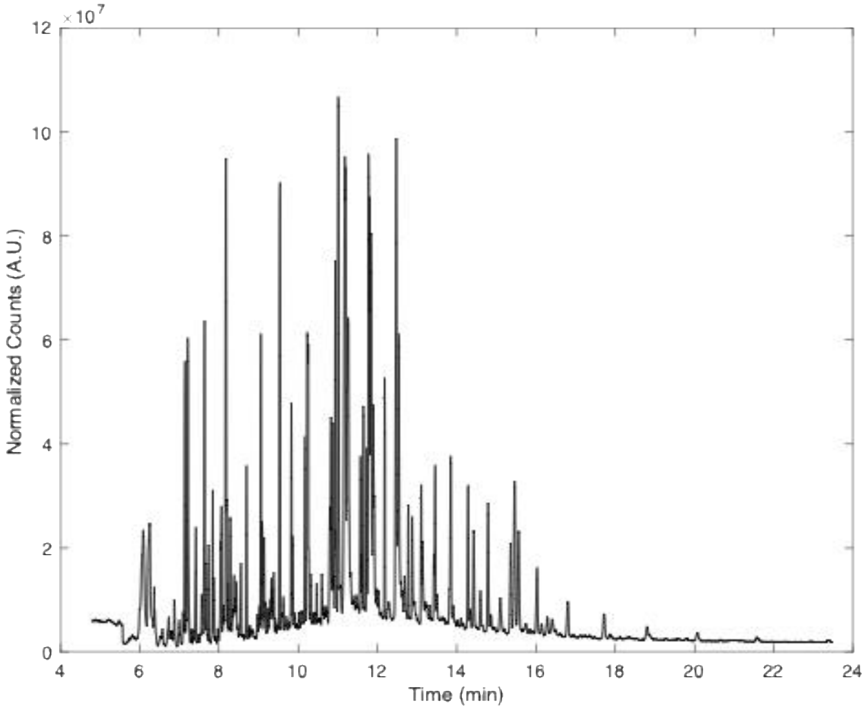

Figure S12. GC-MS chromatogram for Cleaning Liquid 1.

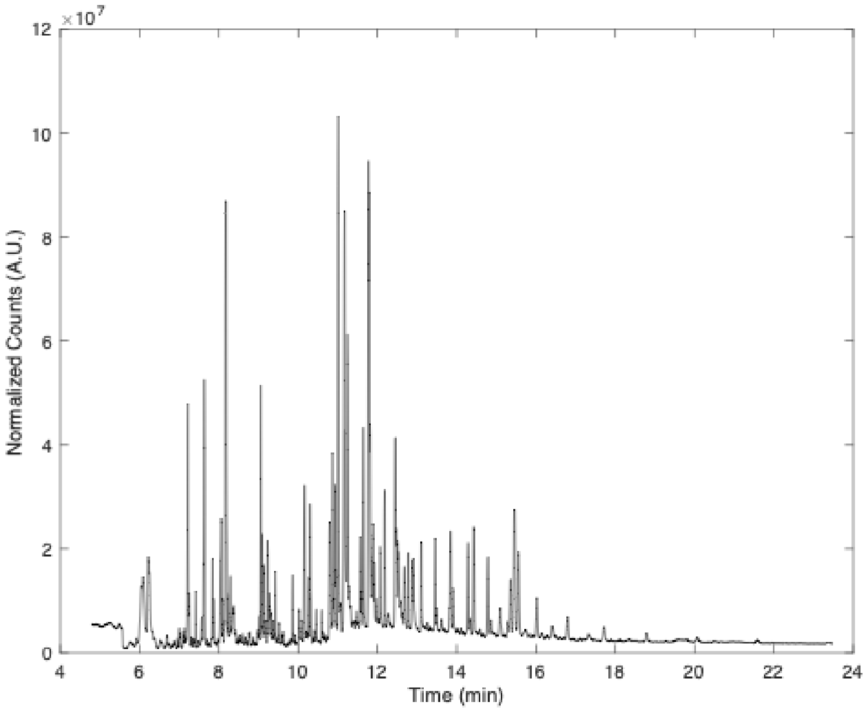

Figure S13. GC-MS chromatogram for Cleaning Liquid 2.

Table S4. Estimated contributions of limonene to total SOA formation, shown in g of limonene SOA formed per gram of product evaporated. These calculations are based on our measured limonene SOA yields for homogeneous and heterogeneous nucleation.

Product	Homogeneous Y_{limSOA} (g g^{-1})	Heterogeneous Y_{limSOA} (g g^{-1})
**Perfume 1**	0.00053	0.00052
**Perfume 2**	0.00117	0.00114
**Deodorant 1**	0.00069	0.00068
**Deodorant 2**	0.00064	0.00062
**Cleaning liquid 1**	0.00122	0.00120
**Cleaning liquid 2**	0.00138	0.00135

Table S5. Percent contributions of limonene to total SOA formed via homogeneous and heterogeneous nucleation. These calculations are based on our measured SOA yields and estimated contributions of limonene to total SOA formation shown in Table S4.

Product	Limonene Contribution to Homogeneous SOA (%)	Limonene Contribution to Heterogeneous SOA (%)
**Perfume 1**	26.5	1.9
**Perfume 2**	23.4	2.8
**Deodorant 1**	2.8	1.1
**Deodorant 2**	5.3	0.4
**Cleaning liquid 1**	20.3	2.3
**Cleaning liquid 2**	1.9	1.1

Table S6. Size-resolved ρ_{eff} values (g cm^{-3}) for SOA formed during homogeneous nucleation experiments. Averages are shown ± standard deviation.

**Product**	**Particle size**		
	**60 nm**	**80 nm**	**100 nm**
**Perfume 1**	1.09 ± 0.01	1.05 ± 0.04	1.09 ± 0.00
**Perfume 2**	1.22 ± 0.05	1.15 ± 0.00	1.08 ± 0.00
**Deodorant 1**	1.65 ± 0.04	1.50 ± 0.03	1.32 ± 0.01
**Deodorant 2**	1.11 ± 0.05	1.18 ± 0.02	1.20 ± 0.00
**Cleaning liquid 1**	1.15 ± 0.04	1.18 ± 0.01	1.17 ± 0.01
**Cleaning liquid 2**	1.46 ± 0.12	1.35 ± 0.02	1.28 ± 0.01
**Limonene**	1.50 ± 0.09	1.44 ± 0.05	1.34 ± 0.03

Table S7. Size-resolved ρ_{eff} values (g cm^{-3}) for SOA formed during heterogeneous nucleation experiments. Averages are shown ± standard deviation.

**Product**	**Particle size**		
	**60 nm**	**80 nm**	**100 nm**
**Perfume 1**	1.51 ± 0.01	1.50 ± 0.01	1.46 ± 0.01
**Perfume 2**	1.53 ± 0.00	1.52 ± 0.00	1.48 ± 0.01
**Deodorant 1**	1.43 ± 0.00	1.47 ± 0.00	1.52 ± 0.01
**Deodorant 2**	1.51 ± 0.01	1.53 ± 0.03	1.52 ± 0.02
**Cleaning liquid 1**	1.48 ± 0.04	1.57 ± 0.01	1.59 ± 0.01
**Cleaning liquid 2**	1.69 ± 0.23	1.59 ± 0.09	1.46 ± 0.01
**Limonene**	1.39 ± 0.07	1.37 ± 0.06	1.39 ± 0.06
**Seed (no SOA)**	1.54 ± 0.02	1.52 ± 0.01	1.48 ± 0.02
